# The association of obstructive sleep apnea and renal outcomes—a systematic review and meta-analysis

**DOI:** 10.1186/s12882-017-0731-2

**Published:** 2017-10-16

**Authors:** Der-Wei Hwu, Kun-Der Lin, Kun-Chen Lin, Yau-Jiunn Lee, Yu-Hung Chang

**Affiliations:** 1 Department of Internal Medicine, Lee’s Endocrinology Clinic, # 130 Min-Tzu Rd, Pingtung, 90000 Taiwan; 2 Division of Endocrinology and Metabolism, Department of Internal Medicine, Kaohsiung Medical University Hospital, Kaohsiung Medical University, No. 100, Tzyou 1st Rd, San-Ming District, Kaohsiung, Taiwan; 3 Graduate institute of Clinical Medicine, Kaohsiung Medical University, Taiwan, No. 100, Shih-Chuan 1st Rd, San-Ming District, Kaohsiung, Taiwan; 4 Department of Internal Medicine, Kaohsiung Municipal Ta-Tung Hospital, Kaohsiung Medical University, Kaohsiung, Taiwan, No.68, Jhonghua 3rd Rd, Cianjin District, Kaohsiung, 80145 Taiwan

**Keywords:** Obstructive sleep apnea, Chronic kidney disease, Diabetes, Proteinuria, Albuminuria

## Abstract

**Background:**

The aim of this systematic review and meta-analysis was to summarize the association of obstructive sleep apnea (OSA) with renal outcome.

**Methods:**

Our study followed the PRISMA guidelines. Two independent reviewers searched for relevant articles in the databases of Pubmed, the Web of Science and CENTRAL, and conducted study selection and quality assessment. A random-effect model was used to estimate the effects.

**Results:**

A total of 1240 articles were initially identified (Pubmed = 568, Web of Science = 640, CENTRAL = 32). After removal of duplicate articles (*n* = 415) and irrelevant articles (*n* = 788), 37 were selected for full-text review, and 18 were finally included in the analysis. Overall, patients diagnosed with OSA were found to have a higher odds ratio (OR) of a poorer renal outcome, with a pooled OR of 1.77 (95% C.I.: 1.37–2.29). The significant association between OSA and a poorer renal outcome was not affected by the medical condition of diabetes mellitus (DM). In addition, we found that OSA was consistently associated with higher albuminuria/proteinuria and a lower estimated glomerular filtration rate (eGFR), with a pooled OR of 1.84 (95% C.I.: 1.24–2.73) and 1.60 (95% C.I.: 1.19–2.16), respectively. A greater OSA severity was also found to be related to a higher OR, with a mild group OR of 1.45 (95% C.I.: 1.19–1.77) and a moderate and severe group OR of 2.39 (95% C.I.: 1.96–2.90).

**Conclusions:**

Our study demonstrated that OSA is significantly associated with poorer renal function.

## Background

According to data from the National Health and Nutrition Examination Survey, the latest report from the United States Renal Data System indicated that chronic kidney disease (CKD) is more common than diabetes mellitus (DM) in the United States, an estimated 13.6% of adults having CKD as compared with 12.3% with DM [1]. Thus, CKD has become a serious public health issue, as it is not only debilitating to individuals, leading to severe complications, but is also a great burden on society, with a large associated cost [2]. Therefore, identifying and disrupting factors that contribute to CKD is not only beneficial to the health of individuals, but can also reduce the cost of treating the disease and its complications.

Obstructive sleep apnea (OSA) could be a potential risk factor for CKD. In the late 1980s, Sklar et al. initially found that OSA could be significantly associated with high-grade proteinuria [3]; they then confirmed these results in a larger cohort [4]. Subsequently, Canales et al. [5] further established that the degree of microalbuminuria is correlated with the severity of OSA. Moreover, Iseki et al. [6] reported that a Japanese population diagnosed with OSA had a higher chance of decreased renal function, as predicted from the estimated glomerular filtration rate (eGFR). Despite these reports suggesting a relationship between OSA and the development of CKD, other conflicting reports have presented evidence against the existence of a connection of OSA with proteinuria [7], albuminuria [8] and the eGFR [5, 9]. Thus, the associations of OSA with diverse renal outcomes should be clarified.

Recently, Leong et al. [10] conducted a meta-analysis that focused on patients with DM, and presented moderate evidence to show that OSA is associated with CKD. However, it should be noted that patients with DM are prone to suffering from nephropathy [2, 11], and the results may not be able to be generalized to other populations. Furthermore, previous studies [10, 12] did not elucidate a relationship between the severity of OSA and the degree of renal function change, which may be worthy of discussion. In order to clarify the uncertainties mentioned above, this meta-analysis study aimed to provide a more comprehensive overview of the relationship between OSA and renal function.

## Methods

Our study followed the PRISMA (Preferred Reporting Items for Systematic Reviews and Meta-Analyses) guidelines [13] during all stages, including design, execution and reporting, when feasible. The present study was registered in the PROPERO database (CRD42015023791).

### Literature search

A systematic literature search was completed at the end of March, 2017. Electronic databases including Pubmed, the Web of Science and the Cochrane Central Register of Controlled Trials (CENTRAL) were searched by two independent investigators (Der-Wei Hwu and Kun-Der Lin). We used a combination of free key words, including “obstructive sleep apnea”, “sleep-disordered breathing”, “chronic kidney disease”, “albuminuria”, “proteinuria”, “renal function” and “nephropathy”, including their MeSH terms, to find relevant articles (see Additional file 1). We also examined published reviews to identify studies that were potentially eligible for inclusion.

### Study selection

We included studies whose participants were aged over 18 years. Articles were excluded based on the following criteria: (1) expert review articles, letters and meeting abstracts; (2) non-English articles; (3) articles that discussed OSA based on a CKD population, for the reason that our aim was to examine renal outcomes in the OSA population. To resolve discrepancies, a consensus was reached with other specialists (Kun-Chen Lin and Yau-Jiunn Lee) who were not involved in the initial search procedure. Study quality was assessed using the Newcastle–Ottawa scale [14].

### Definition of OSA

OSA was defined according to one of the following diagnostic criteria: 1. Apnea-Hypopnea Index (AHI) (as per the American Academy of Sleep Medicine guidance); 2. Oxygen Desaturation Index (ODI); 3. Respiratory Disturbance Index (RDI). Either polysomnography (PSG) or a pulse oximeter was used to make the diagnosis. The diagnostic criteria used in each study are shown in Table 1.

**Table 1:** Summary of the 14 studies included in the meta-analysis

Author/year (country)	Study design	Number of patients	Patient demographics	DM (%)	How renal outcomes and obstructive sleep apnea were evaluated	Main results
Faulx 2007 (US) [31]	Cross-sectional	496	-Cleveland family study -Mean age = 44.5 -Male = 44.4% -Mean BMI = 32.5 kg/m^2^	12.7%	-Renal: ACR (microalbuminuria: 50–250 mg/g) -Sleep tool: PSG OSA severity: low, AHI ≤5; mild, AHI 5–14; moderate, AHI 15–29; severe, AHI ≥ 30	-Significant association between AHI severity and ACR. -ACR level (AHI ≥ 30 vs. control: 7.87 ± 1.02 vs. 5.08 ± 0.41 μg/mg; *P* < 0.006).
Tsioufis 2008 (Greece) [9]	Cross-sectional	132	-Outpatient hypertensive unit -Mean age = 48 -Male = 79.5% -Mean BMI = 32 kg/m^2^	0%	-Renal: ACR (mg/g), eGFR (MDRD) -Sleep tool: PSG OSA severity: AHI ≤ 5, normal; AHI > 5, OSA (+)	-Albuminuria incidence was greater by 57% in OSA patients (ACR: 11 (3~45) vs. 5.6 (0.5~19) mg/g; *P* < 0.001). -eGFR did not differ between the 2 groups (OSA vs. control: 114 ± 30 vs. 116 ± 27 ml/min/1.73m^2^; *P* = 0.6).
Agrawal 2009 (US) [32]	Cross-sectional	91	-Obese patients before bariatric surgery -Mean age = 44.9 -Male = 27.3% -Mean BMI = 48.3 kg/m^2^	34.1%	-Renal: ACR (microalbuminuria: 30–300 mg/g), eGFR (MDRD) -Sleep tool: PSG OSA severity: low, AHI < 5; mild, AHI 5–15; moderate, AHI 16–29; severe, AHI ≥ 30	-ACR did not differ between OSA group vs. control group: 8 (5~16) vs. 6(4~14.5) μg/mg; *P* = 0.723.
Laaban 2009 (France) [33]	Cross-sectional	303	-Hospitalized poorly-controlled T2DM patients -Mean age = 61.3 -Male = 51.5% -Mean BMI = 32.0 kg/m^2^	100%	-Renal: microalbuminuria (>30 mg/24 h) -Sleep tool: nocturnal respiratory polygraphic study using analyses of nasal airflow, tracheal sounds and oximetry OSA severity: normal, RDI < 5; mild, RDI 5–15; moderate, RDI 16–29; severe, RDI ≥ 30	-Prevalence of microalbuminuria did not differ between the controls and each OSA group (control vs. mild vs. moderate vs. severe: 25% vs. 34% vs. 38% vs. 35%; *P* > 0.05).
Canales 2011 (US) [5]	Cross-sectional	507	- Community study -Mean age = 76 -Male = 100% -Mean BMI = 27.9 kg/m^2a^	13%	-Renal: ACR (clinical albuminuria >30 mg/gCr) -Sleep tool: portable PSG OSA severity: normal, RDI 0~4.9; mild, RDI 5–14.9; moderate, RDI 15–29.9; severe, RDI ≥ 30	-Graded association between RDI and ACR (RDI ≥ 30 vs. control: 9.35 vs. 6.72, *P* = 0.007). -eGFR did not differ between the 4 groups (control: 70.4 ± 13.6; mild: 70.7 ± 15.7; moderate: 69.6 ± 15.3; severe: 67.9 ± 14.0 ml/min/1.73m^2^; *P* = 0.55).
Buyukaydin 2012 (Turkey) [8]	Cross-sectional	52	-Mean age = 56 -Male = 27% -Mean BMI = 32.4 kg/m^2^	100%	-Renal: ACR (microalbuminuria: 30–300 mg/g) -Sleep tool: PSG OSA severity: low, AHI < 5; mild, AHI 5–15; moderate, AHI 16–30; severe, AHI > 30	-No significant relationships between respiratory obstructive parameters and microalbuminuria (*R* = 0.91, *P* = 0.362).
Kanbay 2012 (Turkey) [34]	Cross-sectional	175	-Patients referred for sleep tests -Mean age = 53.7^a^ -Male = 66.9% -Mean BMI = 31.8 kg/m^2^	24.2%	-Renal: eGFR (Cockcroft–Gault formula) -Sleep tool: PSG OSA severity: normal AHI < 5; mild, AHI 5–15; moderate, AHI 15–30; severe, AHI > 30	-Decrease in the eGFR noted when the severity of OSA increased (control: 50 ± 11.8; mild: 44.8 ± 15.7; moderate: 40.8 ± 14.7; severe: 38.8 ± 15.9 ml/min/1.73m^2^; *P* < 0.001).
Furukawa 2013 (Japan) [35]	Cross-sectional	513	-From the Dogo Study -Mean age = 62.0 -Male = 56.9% -Mean BMI = 25.2 kg/m^2^	100%	-Renal: ACR (microalbuminuria, ≥3.4 mg/mmol creatinine; nephropathy, ≥34 mg/mmol creatinine) -Sleep tool: pulse oximeter OSA severity: 3% ODI (normal, ODI < 5; NH, ODI ≥5)	-NH may be an independent risk factor for albuminuria (more significant in female patients). -NH and microalbuminuria OR = 1.84 (95% C.I.:1.16–2.96). -NH and nephropathy (macroalbuminuria) OR = 2.97 (95% C.I.:1.36–6.90).
Sakaguchi 2013 (Japan) [36]	Retrospective	161	-Patients with CKD stage 3 or 4 -Mean age = 68.8 -Male = 75.8% -Median BMI = 21.8 kg/m^2^	24.2%	-Renal: eGFR (equation for Japanese populations) -Sleep tool: pulse oximeter OSA severity: 4% ODI (normal, ODI < 5; mild, 5 ≤ ODI < 15; moderate-to-severe, 15 ≤ ODI)	-The eGFR declined faster in patients with moderate-to-severe NH than in patients with no or mild NH. -Mean values (95% C.I.) for eGFR decline: control: −2.14 (−1.06- ~3.21); mild: −3.02 (−1.31~ −4.74]; moderate & severe: −8.59 (−2.00 ~ −15.2) ml/min/1.73m^2^; *P* = 0.003.
Tahrani 2013 (UK) [37]	Prospective	224	-Mean age = 56.6^a^ -Male = 53.5% -Mean BMI = 33.5 kg/m^2^	100%	-Renal: ACR (microalbuminuria >3.4 mg/mmol; macroalbuminuria >30 mg/mmol), eGFR (MDRDS) -Sleep tool: portable PSG OSA severity: normal, AHI <5; mild, AHI 5–15; moderate, AHI 16–29; severe, AHI ≥ 30	-Cross-sectional association of OSA and CKD: OR = 2.64 (95% C.I.: 1.13~6.16). -AHI is a predictor of the study-end eGFR.
Leong 2014 (UK) [38]	Cross-sectional	90	- Obese patients referred to a weight management service -Mean age = 51 -Male = 43% -Mean BMI = 46.8 kg/m^2^	100%	-Renal: eGFR (CKD-EPI) -Sleep tool: portable PSG OSA severity: AHI < 5, normal; AHI ≥ 5, OSA (+)	-Apnea and hypopnea events, as well as the duration of NH, were inversely associated with renal function after adjusting for potential confounders.
Storgaard 2014 (Denmark) [39]	Cross-sectional	200	-Mean age = 59.6 -Male = 61% -Mean BMI = 31.7 kg/m^2^	100%	-Renal: UACR (microalbuminuria: 30–300 mg/g; macroalbuminuria: ≥ 300 mg/g) -Sleep tool: PSG OSA severity: low, AHI <5; mild, 5 ≤ AHI < 15; moderate, 15 ≤ AHI ≤ 30; severe, AHI > 30	-There were no obvious differences between the OSA (+) and OSA (−) groups regarding micro/macro-proteinuria (*P* = 0.2).
Bulcun 2015 (Turkey) [40]	Cross-sectional	124	-Patients referred for sleep tests -Mean age = 47.1^a^ -Male = 74.2% -Mean BMI = 31.3 kg/m^2a^	0%	-Renal: ACR (microalbuminuria/creatinine ratio: 20–299 mg/g), eGFR (MDRD) -Sleep tool: PSG OSA severity: AHI < 5, non-apneic; AHI ≥ 5, OSA(+)	-OSA is positively associated with UACR level (control: 8.2 ± 12.7; OSA: 25.5 ± 51.4 mg/g, *p* = 0.004), while the eGFR level showed no clinical significant differences.
Zhang 2015 (China) [42]	Cross-sectional	472	-Hospitalized poorly-controlled T2DM patients -Mean age = 55 -Male = 68% -Mean BMI = 26.5 kg/m^2^	100%	-Renal: ACR (microalbuminuria/creatinine ratio ≥ 300 mg) or based on a medical history of diabetic nephropathy -Sleep tool: PSG OSA severity: low, AHI <5; mild, AHI ≥5; moderate, AHI ≥15; severe, AHI ≥ 30	-High prevalence of OSA in this population (66.7%). -No association between OSA and diabetic nephropathy
Chang 2016 (Taiwan) [43]	Cross-sectional	988	-Patients that had undergone PSG -Mean age = 51.1 -Male = 71.4% -Mean BMI = 26.7 kg/m^2^	15.6%	-Renal: eGFR (CKD-EPI) -Sleep tool: PSG OSA severity: low, AHI <5; mild and moderate, 5 ≤ AHI < 30; severe, AHI ≥ 30	-The multivariable odds ratio of CKD was highest in patients with both resistant hypertension and severe sleep apnea (OR: 13.42; 95% C.I.: 4.74–38.03; *P* < 0.001).
Uyar 2016 (Turkey) [41]	Cross-sectional	696	-Patients referred for sleep tests -Mean age = 50.4^a^ -Male = 68.1% -Mean BMI = 32.0 kg/m^2a^	NA Mean blood glucose: OSA:112 mg/dl Control:103 mg/dl	-Renal: eGFR (CKD-EPI) -Sleep tool: PSG OSA severity: low, AHI <5; mild, AHI, 5–15; moderate, AHI 16–29; severe, AHI ≥ 30	-No association between OSA and the eGFR (eGFR: control 94.14 ± 18.81; OSA 90.73 ± 19.59 ml/min/1.73m^2^, *P* = 0.19).
Zhang 2016 (China) [44]	Cross-sectional	880	-Hospitalized patients -Mean age = 59.2 -Male = 55.6% -Mean BMI = 25.1 kg/m^2^	100%	-Renal: ACR (microalbuminuria/creatinine ratio), eGFR (MDRD); classified into 3 stages: microalbuminuria, macroalbuminuria and renal insufficiency -Sleep tool: PSG OSA severity: AHI, ODI, cumulative duration of SPO_2_ below 90% and 85%	-The cumulative duration of SPO2 below 90% was independently associated with diabetic nephropathy. -Macroalbuminuria and renal insufficiency did not have significant associations with diabetic nephropathy.
Adams 2017 (Australia) [45]	Cross-sectional	986 (812 were able to finish PSG)	-Mean age = NA -Male = 100% -Mean BMI = NA	<20%	-Renal: ACR (microalbuminuria/creatinine ratio), eGFR (CKD-EPI) Sleep tool: PSG OSA severity: low, AHI <10; mild, AHI 10–19; moderate, AHI 20–29; severe, AHI ≥ 30	-CKD of predominantly mild severity (stage 1–3) showed significant associations with OSA. AHI ≥ 10: OR = 1.9 (95% C.I.: 1.02–3.5); AHI ≥ 30: OR = 2.6 (95% C.I.: 1.1–6.2).

*T2DM* type 2 diabetes mellitus, *PSG* polysomnography, *eGFR* estimated glomerular filtration rate, *MDRD* modification of diet in renal disease study equation, *AHI* Apnea-Hypopnea Index, *DN* diabetic nephropathy, *ODI* oxygen desaturation index, *NH* nocturnal hypoxia, *ACR* albumin creatinine ratio, *RDI* respiratory distress index, *CKD-EPI* chronic kidney disease- Epidemiology Collaboration equation, *NA* not available

AHI = count of the number of apneas and hypopneas per hour of sleep

RDI = total number of apnea and hypopnea events per hour of recording

3% ODI = the total number of events during which a person’s oxygenation dropped >3% in an hour

^a^calculated average value

### Renal outcomes

We included studies that reported renal outcomes according to the status of albuminuria/proteinuria or the eGFR. Albuminuria/proteinuria was defined by the urinary albumin/protein to creatinine ratio. As there are various methods for calculating the eGFR (e.g., MDRD, CKD-EPI), we included eligible studies as long as their methods were described clearly.

### Data synthesis and analysis

Data were extracted using a standardized data extraction form. General information regarding the study (e.g., author, publication year, and study design) and the characteristics of the study population (e.g., age, body mass index, and percentages of subjects with DM, hypertension and CKD) were extracted. For studies that examined more than one of our populations of interest (e.g., DM and non-DM; mild, moderate and severe OSA) or renal outcomes (albuminuria/proteinuria and eGFR) by subgroup analysis, each result was treated as an independent study in our analysis. For longitudinal studies, we only extracted the before–after changes in renal outcomes, which are more convincing than cross-sectional data. For studies that lacked important numeric values, we attempted to contact the original authors to obtain the missing data.

We used a random-effect model to conduct our meta-analysis owing to the significant heterogeneities among the included studies (e.g., variations in study design, study population, measurements of OSA and renal outcomes). If the data were presented as medians instead of averages, and ranges or quartiles instead of standard deviations, they were first converted into averages and standard deviations using the equations provided by Hozo et al. [15]. In each meta-analysis, if multiple data entries were presented in one study, we pooled these data into a single data entry in the analysis via the equations provided by Borenstein et al. [16]. Owing to the fact that our preliminary data included binary outcomes and continuous variables, we transformed the continuous variables into ORs (see Additional file 2) in order to make our results less complicated and easier to understand.

We discussed the overall relationship of OSA with renal function, and presented subgroup results by study design, verifying the results of Leong et al. [10] via subgroup analysis of diabetes status. For studies that included a population of mixed diabetes status, we considered those with a DM population < 40% as non-DM studies. The reason for choosing 40% as the cut-off point was that clinical information obtained from a subgroup consisting of less than 40% of the study population may lack clinical significance, as explained by Wittes et al. [17]. We also performed subgroup analysis according to the eGFR, proteinuria/albuminuria and OSA severity in order to clarify the association between OSA and renal function. Odds ratios (ORs) were used to present the relationship between OSA and CKD. Publication bias was assessed by Egger’s test. All of the analyses were performed using Comprehensive Meta-Analysis 2.0 software (Biostat, Englewood, NJ, https://www.meta-analysis.com/). We considered a *P*-value of less than 0.05 to indicate statistical significance.

## Results

**Fig. 1:**
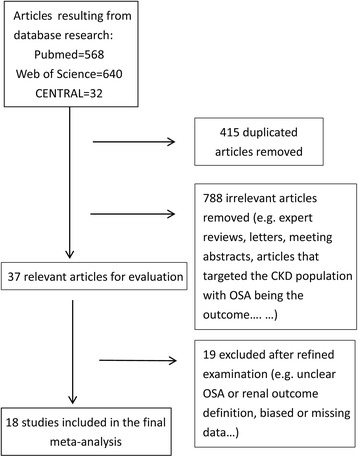
Flow chart of article extraction for meta-analysis

A flow diagram of the search for relevant trials is presented in Fig. 1. In total, 1240 studies were initially identified (Pubmed = 568, Web of Science = 640, CENTRAL = 32). After removal of duplicate articles (*n* = 415) and irrelevant articles (*n* = 788), 37 articles were selected for full-text review. A further 19 articles were excluded following detailed review, of which 3 were review articles, which we reviewed to find potential relevant articles [10, 12, 18]. The remaining 16 were excluded for the following reasons: 1 focused on central apnea [19], 6 did not have a clear definition of nephropathy [20–25], 3 may have contained biased data (e.g., 1 related to creatinine clearance, i.e., over 200 ml/min [26], 2 had fewer than 10 subgroup participants [27, 28]), 3 had missing data [6, 7, 29], and 1 focused only on the OSA population without a control group [30]. Finally, 18 studies [5, 8, 9, 31–45] that included 7090 patients were incorporated in our meta-analysis.

### Study characteristics

A summary of the 18 studies is presented in Table 1. Two of the 18 studies were longitudinal [36, 37], the rest being cross-sectional [5, 8, 9, 31–35, 38–45]. Three of the studies were performed in the US [5, 31, 32], 2 in Japan [35, 36], 1 in Taiwan [43], 2 in China [42, 44], 1 in Australia [45], and the rest in Europe [8, 9, 33, 34, 37–41]. Fourteen studies used the AHI to diagnose OSA, while OSA was defined using the ODI [35, 36] or RDI [5, 33] in the remaining studies.

### Meta-analysis results

**Fig. 2:**
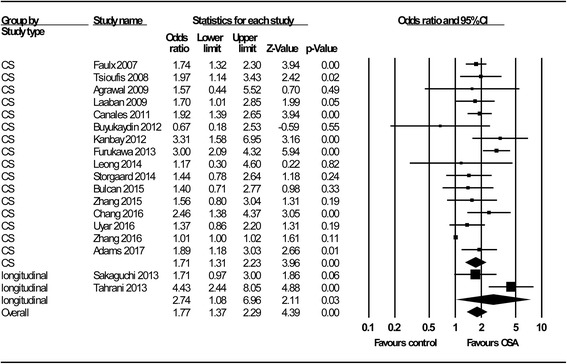
Meta-analysis results regarding the impact of OSA on CKD

**Fig. 3:**
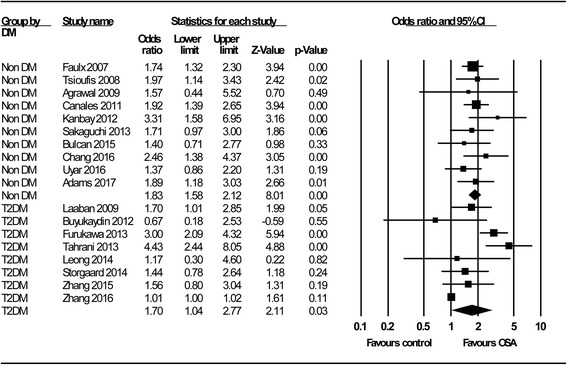
Subgroup analysis by diabetes status

**Fig. 4:**
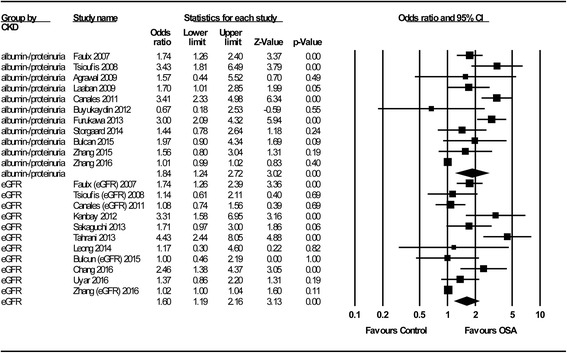
Subgroup analysis by renal outcomes

**Fig. 5:**
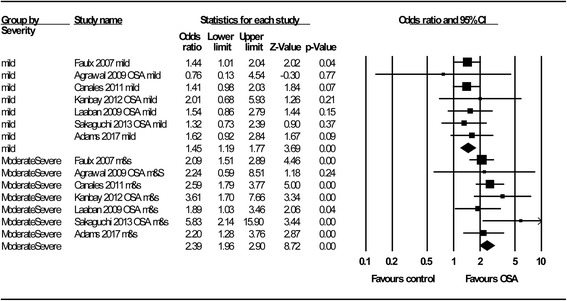
Subgroup analysis by OSA severity

The overall association of OSA with renal function is shown in Fig. 2. We identified a significant relationship between OSA and poorer renal function, with a pooled OR of 1.77 (95% C.I.: 1.37–2.29; *P* < 0.001); this association did not change significantly with different study designs (OR = 1.71, 95% C.I.: 1.31–2.23; *P* < 0.001 for cross-sectional studies; OR = 2.74, 95% C.I.: 1.08–6.96; *P* = 0.03 for longitudinal studies). By subgroup analysis (Fig. 3), we not only confirmed the results of the study by Leong et al. [10], which reported that OSA is significantly associated with poorer renal function in patients with DM (OR = 1.70; 95% C.I.: 1.04–2.77; *P* = 0.03), but also expanded this association to patients without DM (OR = 1.83; 95% C.I.: 1.58–2.12; *P* < 0.001). OSA was associated with adverse renal outcomes, whether represented by albuminuria/proteinuria (OR = 1.84; 95% C.I.: 1.24–2.72; *P* < 0.001) or by the eGFR (OR =1.60; 95% C.I.: 1.19–2.16; *P* < 0.001) (Fig. 4). In addition, we demonstrated for the first time that impaired renal outcome could occur even in patients with mild OSA (OR = 1.45; 95% C.I.: 1.19–1.77; *P* < 0.001), and the numeric value of the OR was higher in patients with moderate or severe OSA, which may be taken as indirect evidence showing that OSA acts as a contributing factor to a poorer renal outcome (OR = 2.39; 95% C.I.: 1.96–2.90; *P* < 0.001) (Fig. 5).

With regards to all analyses, no publication bias was found by Egger’s test. No studies in individual populations dramatically influenced the overall results according to the outcomes of the remove-one-study sensitivity test in the meta-analysis.

### Quality assessment

Table 2 summarizes the results in terms of study quality. The overall quality scores were averaged, with 8 studies rated moderate, 6 studies rated strong, and 4 rated weak. The blinding component and study design component had the lowest ratings.

**Table 2:** Newcastle–Ottawa scale for quality assessment

Study	Selection bias	Respiratory measurement	Blinding	Study design	Analysis	Overall
Faulx 2007 [31]	Moderate	Strong	Weak	Weak	Strong	Moderate
Tsioufis 2008 [9]	Strong	Strong	Weak	Weak	Strong	Strong
Agrawal 2009 [32]	Moderate	Strong	Strong	Weak	Strong	Strong
Laaban 2009 [33]	Moderate	Moderate	Weak	Weak	Moderate	Moderate
Canales 2011 [5]	Weak	Moderate	Weak	Weak	Strong	Weak
Buyukaydin 2012 [8]	Weak	Strong	Weak	Weak	Weak	Weak
Kanbay 2012 [34]	Moderate	Strong	Weak	Weak	Moderate	Weak
Furukawa 2013 [35]	Weak	Moderate	Weak	Weak	Strong	Weak
Sakaguchi 2013 [36]	Weak	Strong	Weak	Moderate	Moderate	Moderate
Tahrani 2013 [37]	Weak	Strong	Weak	Strong	Strong	Strong
Leong 2014 [38]	Strong	Strong	Strong	Moderate	Strong	Strong
Storgaard 2014 [39]	Strong	Strong	Weak	Weak	Weak	Moderate
Bulcun 2015 [40]	Strong	Strong	Weak	Moderate	Strong	Strong
Zhang 2015 [42]	Moderate	Moderate	Weak	Weak	Moderate	Moderate
Chang 2016 [43]	Strong	Strong	Weak	Weak	Strong	Strong
Uyar 2016 [41]	Strong	Strong	Weak	Weak	Moderate	Moderate
Zhang 2016 [44]	Strong	Moderate	Weak	Moderate	Strong	Moderate
Adams 2017 [45]	Moderate	Strong	Weak	Weak	Strong	Moderate

## Discussion

Our results indicated that OSA is not only significantly associated with poorer renal function in patients with DM, but also in patients without DM. In addition, we consolidated the significance of OSA in poorer renal function by reporting on different study designs, renal outcomes (eGFR or albuminuria/proteinuria) and OSA severities.

A recent meta-analysis by Leong [10] demonstrated that OSA is associated with poorer renal function in the diabetic population. However, the nature of diabetic patients leaves them more prone to suffering from nephropathy [2, 11], and therefore it could be suspected that this association may not apply to populations without DM. However, in our analysis, we not only confirmed that OSA is significantly associated with poorer renal function in diabetic patients, but also found that this relationship may also apply to non-diabetic patients. Thus, healthcare providers should be aware of the symptoms of OSA in clinical practice.

Possible mechanisms of the interaction between OSA and renal function change have been proposed. Turek et al. [12] pointed out that a specific feature of OSA is intermittent hypoxia followed by reoxygenation, stimulating the formation of reactive oxygen species, which promote inflammation and systemic endothelial dysfunction. During the hypoxia period, the rise in sympathetic tone and activation of the renin-angiotensin system cause the systematic and intraglomerular pressure to rise [46]. Therefore, it is plausible that the risk of CKD increases in parallel with an increased hypoxia duration and greater frequency, as confirmed in the meta-analysis examining the relationship of OSA severity with renal outcome. Of note, our results showing that there is an increased risk of CKD even in patients with mild OSA may be of particular importance, as there has been disagreement regarding treatment for patients with mild OSA [47, 48]. Thus, our result may present a different perspective on the clinical management of patients with OSA.

Despite our results suggesting a close relationship between OSA and poorer renal function, and implying that medical therapy that alleviates OSA could slow the development of CKD, clear evidence is still lacking. In a subgroup population with moderate-to-severe OSA, Tahrani et al. [37] demonstrated that participants adherent to continuous positive airway pressure (CPAP) therapy exhibited a slower decline in renal function as compared with CPAP non-adherent patients (−7.7% vs. -10%). In a retrospective study, Puckrin et al. [49] reported that participants who underwent CPAP therapy more often had lesser renal function decline and proteinuria. Despite these reports indicating a promising utility of CPAP for renal function protection in OSA patients, the results are limited by the small number of participants (< 50) [37, 49], a lack of statistical significance [37], and potential confounders in a retrospective study design [49]. In addition, these reports also truthfully reflect the limitations of CPAP in clinical practice, as most patients under OSA management have been found to be non-adherent (>60%) to CPAP [37, 49]. In the context that none of these reports showed any renal benefit of CPAP in mild OSA patients [37], further investigation may be necessary to ascertain the benefits of OSA management in CKD patients.

One of the purposes of this meta-analysis was to resolve discrepancies between some studies concerning the association between OSA and CKD. In a report based on 507 community-dwelling elderly men, Canales et al. showed that the severity of albuminuria increased with increasing severity of OSA, but the eGFR did not decrease with increasing severity of OSA [5]. Likewise, in a study that included 132 patients with untreated hypertension, Tsioufis et al. found that patients with OSA exhibited a higher degree of albuminuria, but there was no difference in renal function as estimated from the eGFR [9]. These reports may confuse the association between OSA and CKD. However, it should be noted again that albuminuria is an early indicator of renal damage, and a longer duration may be needed in order to observe a change in the eGFR. In addition, these results may be hampered by the limitations of the cross-sectional design, in terms of not being able to provide a clear chain of consequence between OSA and CKD. In contrast to these two studies, in a prospective cohort study based on 224 type 2 DM (T2DM) patients, Tahrani et al. [37] not only showed that in patients with OSA, the prevalence of diabetic nephropathy at baseline was significantly higher, but also clearly indicated that T2DM patients with OSA exhibited a greater eGFR decline at the end of the 2.5-year follow-up period. Taking these results together with the results of our meta-analysis, we concluded that OSA is significantly associated with poorer renal function, whether defined by albuminuria/proteinuria or the eGFR.

There were several limitations in our study. First, among the 18 eligible studies, only 2 cohort studies discussed the impact of OSA on renal function change [36, 37]. Of note, only one study was of a prospective design [37], the other being retrospective in nature [36]. Owing to study results potentially being biased by the nature of the study design, our results should be interpreted with caution. Second, it has been reported that there is an approximate 40% variation in the measurement of albuminuria [50], and significant inconsistency exists among various eGFR formulas [51]. Thus, our results may be biased by the renal function measurements obtained in these studies. Third, to maximize the information available, we artificially transformed the renal outcomes reported in some studies into odds ratios, which may have overinflated the results. Also, in most of included studies, unadjusted analyses were performed, and residual confounders remained. Thus, our results should be interpreted carefully. Fourth, our results may also be biased by the different diagnostic tools used to define OSA; they also may not apply to young adults, as most of the included study populations were aged over 50. Despite these limitations, our study is of merit in terms of showing a consistent and clear association between OSA and CKD from different perspectives. In addition, compared with other articles [10], our results were based on more sophisticated evidence in terms of not including potentially biased reports (e.g., including meeting abstracts [52, 53], studies with an unclear renal outcome definition, etc. [21]). Based on the above discussion, our results may provide additional information relevant to clinical practice.

## Conclusions

In conclusion, our results indicated that OSA was significantly related to poorer renal function, suggesting that diagnosis of OSA should not be overlooked in clinical practice. Larger prospective studies may be necessary in order to compare renal outcomes with/without medical intervention to provide more persuasive data regarding the causality between OSA and renal function, particularly in patients with mild OSA.

## Additional files

Additional file 1: Search terms for Pubmed, Web of Science and CENTRAL databases. (DOCX 13 kb)

Additional file 2: Transforming continuous variables into odds ratios (ORs). (DOCX 22 kb)

## Abbreviations

AHI: Apnea-Hypopnea Index
CENTRAL: Cochrane Central Register of Controlled Trials
CKD: Chronic kidney disease
CPAP: Continuous positive airway pressure
DM: Diabetes mellitus
eGFR: Estimated glomerular filtration rate
ODI: Oxygen Desaturation Index
OR: Odds rations
OSA: Obstructive sleep apnea
PRISMA: Preferred Reporting Items for Systematic Reviews and Meta-Analyses
PSG: Polysomnography
RDI: Respiratory Disturbance Index
T2DM: Type 2 DM
