# Supplementary material for: The association of obstructive sleep apnea and renal outcomes—a systematic review and meta-analysis
Source: BMC Nephrol. 2017 Oct 16;18:313. doi: 10.1186/s12882-017-0731-2 (PMC5644098; doi:10.1186/s12882-017-0731-2)
Supplement: Supplementary file 1 — Search terms for Pubmed, Web of Science and CENTRAL databases. (DOCX 13 kb) [file 12882_2017_731_MOESM1_ESM.docx]

**Additional file 1: Search terms for Pubmed, Web of Science and CENTRAL databases**

| **Search strategy** |
| --- |
| 1. obstructive sleep apnea [MeSH] 2. sleep-disordered breathing [MeSH] 3. 1 or 2 4. chronic kidney disease [MeSH] 5. albuminuria [MeSH] 6. proteinuria [MeSH] 7. nephropathy [MeSH] 8. renal function [MeSH] 9. 3 or 4 or 5 or 6 or 7 or 8 10. 3 and 9 |
