# Supplementary material for: The association of obstructive sleep apnea and renal outcomes—a systematic review and meta-analysis
Source: BMC Nephrol. 2017 Oct 16;18:313. doi: 10.1186/s12882-017-0731-2 (PMC5644098; doi:10.1186/s12882-017-0731-2)
Supplement: Supplementary file 2 — Transforming continuous variables into odds ratios (ORs). (DOCX 22 kb) [file 12882_2017_731_MOESM2_ESM.docx]

**Additional file 2: Transforming continuous variables into odds ratios (ORs)**

$\overline{X}_{G1}-\overline{X}_{G2}$: difference in mean outcome between groups

*s_pooled_*: standard deviation of outcome among participants

$\overline{ES}$: standard mean difference

Log odds ratio: PI*$\overline{ES}$/√3

OddsRatio = Exp(Log Odds Ratio)

PI=3.14159265358979
